# Hsa-miR-125a-3p and hsa-miR-125a-5p are downregulated in non-small cell lung cancer and have inverse effects on invasion and migration of lung cancer cells

**DOI:** 10.1186/1471-2407-10-318

**Published:** 2010-06-22

**Authors:** Lili Jiang, Qin Huang, Siyang Zhang, Qingfu Zhang, Jihong Chang, Xueshan Qiu, Enhua Wang

**Affiliations:** 1 Department of Pathology, the First Affiliated Hospital of China Medical University, No. 92 North Second Road, Heping District, Shenyang, Liaoning 110001, China; 2 Department of Pathology, College of Basic Medical Sciences, China Medical University, No. 92 North Second Road, Heping District, Shenyang, Liaoning 110001, China; 3 Center of Laboratory Technology and Experimental Medicine, China Medical University, No. 92 North Second Road, Heping District, Shenyang, Liaoning 110001, China

## Abstract

**Background:**

Two mature microRNAs (miRNAs), hsa-miR-125a-3p and hsa-miR-125a-5p (collectively referred to as hsa-miR-125a-3p/5p), are derived from 3' and 5' ends of pre-miR-125a, respectively. Although impaired regulation of hsa-miR-125a-5p has been observed in some tumors, the role of this miRNA in invasion and metastasis remains unclear, and few studies have examined the function of hsa-miR-125a-3p. In order to characterize the functions of hsa-miR-125a-3p/5p in invasion and metastasis of non-small cell lung cancer (NSCLC), we investigated the relationships between hsa-miR-125a-3p/5p expression and lymph node metastasis in NSCLC tissues. We also explored the impact of expression of these miRNAs on invasive and migratory capabilities of lung cancer cells.

**Methods:**

Expression of hsa-miR-125a-3p/5p in NSCLC tissues was explored using real-time PCR. The relationships between hsa-miR-125a-3p/5p expression and pathological stage or lymph node metastasis were assessed using the Spearman correlation test. For in vitro studies, lung cancer cells were transfected with sense and antisense 2'-O-methyl oligonucleotides for gain-of-function and loss-of-function experiments. Transwell experiments were performed to evaluate cellular migration and invasion.

**Results:**

Expression of hsa-miR-125a-3p/5p was lower in NSCLC tissues than in adjacent normal lung tissues (LAC). Furthermore, the results from the Spearman correlation test showed a negative relationship between hsa-miR-125a-3p expression and pathological stage or lymph node metastasis and an inverse relationship between hsa-miR-125a-5p expression and pathological stage or lymph node metastasis. In vitro gain-of-function experiments indicated that hsa-miR-125a-3p and hsa-miR-125a-5p function in an opposing manner, suppressing or enhancing cell migration and invasion in A549 and SPC-A-1 cell lines, respectively. These opposing functions were further validated by suppression of hsa-miR-125a-3p and hsa-miR-125a-5p expression in loss-of-function experiments.

**Conclusion:**

Hsa-miR-125a-3p and hsa-miR-125a-5p play distinct roles in regulation of invasive and metastatic capabilities of lung cancer cells, consistent with the opposing correlations between the expression of these miRNAs and lymph node metastasis in NSCLC. These results provide new insights into the roles of miR-125a family members in the development of NSCLC.

## Background

MicroRNAs (miRNAs) are a class of endogenous, noncoding RNAs, approximately 20-24 nucleotides in length, that are derived from longer transcripts termed pri-miRNAs and pre-miRNAs [1-5]. MiRNAs recognize target mRNAs through partial complementarity to specific sequences within the mRNAs and posttranscriptionally regulate gene expression in multicellular organisms [6-9]. Emerging evidence has shown that human miRNA genes are frequently located in cancer-associated genomic regions, and perturbed miRNA expression patterns have been detected in many human cancers [10]. Therefore, it is of utmost importance to further elucidate the biological functions of miRNAs.

Recently, miRNAs have been shown to play a role in invasion and metastasis [11-15]. For example, miR-155 may play an important role in the TGF-β-induced epithelial-mesenchymal transition (EMT) and in cell migration and invasion through targeting of the RhoA transcript [16]. MiR-21 has been shown to stimulate cell invasion and metastasis in several tumor models, including breast cancer [12], colon cancer [17], and glioma [18]. MiR-10b can be activated by the pro-metastatic transcription factor TWIST1 and is essential for TWIST1-induced EMT involved in promotion of cell motility and invasiveness [19]. Tumor invasion and metastasis are the critical steps that define the prognosis of cancer patients. Therefore, understanding the specific roles of miRNAs in cancer progression could lead to the identification of predictive markers and the development of novel therapeutic strategies for patients with metastases.

MiR-125a is one of the many miRNAs that remain to be fully characterized. Using miRNA microarray analysis, Yanainhara and colleagues [20] found that miR-125a, specifically the hsa-miR-125a-5p mature miRNA, is located at 19q13.41 and that its expression is downregulated in NSCLC. Recently, a new member of the mature miR-125a family has been identified and named hsa-miR-125a-3p. Unfortunately, the expression and function of hsa-miR-125a-3p are currently unknown. In this study, we found that expression of both hsa-miR-125a-5p and hsa-miR-125a-3p is decreased significantly in NSCLC tissues in comparison to LAC tissues. Changes in expression of both hsa-miR-125a-3p and hsa-miR-125a-5p are associated with pathological stage and lymph node metastasis in lung cancer, but in an opposing manner as shown by the Spearman correlation test. In cellular studies, hsa-miR-125a-3p and hsa-miR-125a-5p also appeared to function in opposing manners in lung cancer cells, suppressing or enhancing cell migration and invasion, respectively. These results identify a potential role for the miR-125a family in metastasis of NSCLC.

## Methods

### Samples

We analyzed 52 pairs of non-small cell lung cancer specimens and corresponding normal lung tissues (LAC) collected at the time of surgery and prior to chemotherapy. Specimens were obtained from patients at the First Affiliated Hospital of China Medical University from 1 January 2006 to 1 December 2007 with informed consent. For the majority of samples, clinical and biological information was available. The study has been approved by the Hospitals' Ethical Review Committee.

### Cell culture

The HBE (Human Bronchiolar Epithelium) cell line and the human lung cancer cell lines SPC-A-1 (adenocarcinoma), LH7 and NCI-H460 (large cell cancinoma) were propagated in RPMI1640 (Gibco). The A549 (adenocarcinoma) cell line was propagated in Dulbecco's Modifed Eagle Medium (Gibco). In both cases, the medium was supplemented with 10% fetal bovine serum (FBS), 100 U/ml penicillin, and 100 U/ml streptomycin. Cells were cultured at 37°C in 5% CO_2 _until they reached a confluency of 75%.

### Transfection

Depletion of hsa-miR-125a-3p/5p in the A549 and SPC-A-1 cell lines was achieved through transfection with antisense 2'-O-methyl oligonucleotides directed against hsa-miR-125a-3p/5p. Cells were transfected using Lipofetamine™ 2000 (Invitrogen) according to the manufacturer's protocol.

Briefly, complexes containing the oligonucleotides were prepared according to the recommended protocol and added directly to cells at a final oligonucleotide concentration of 0.4 nmol/mL. Oligonucleotides composed entirely of 2'-O-methyl bases were chemically synthesized by Integrated DNA Technologies (GeneChem) and were comprised of the following sequences: 2'-O-Me-sense-3p: 5'-ACA GGU GAG GUU CUU GGG AGCC-3' 2'-O-Me-antisense-3p: 5'-GGC UCC CAA GAA CC U CAC CUGU-3', 2'-O-Me-scramble-3p: 5'-GGU CGG UGC UCG AUG CAG GUAA-3', 2'-O-Me-sense-5p: 5'-UCC CUG AGA CCC UUU AAC CUG UGA-3', 2'-O-Me-antisense- 5p: 5'-UCA CAG GUU AAA GGG UCU CAG GGA-3', 2'-O-Me-scramble-5p: 5'-GGA CG G CGA UCA GAU AAG AGU UCU-3'. Cells were divided into four groups: an untreated group incubated only in the normal media (untreated), a group transfected with the scrambled 2'-O-methyl oligonucleotide (scramble); a group transfected with the sense 2'-O-methyl oligonucleotide (sense) and a group transfected with the antisense 2'-O-methyl oligonucleotide (antisense). The untreated and scramble groups served as negative controls.

### Quantitative real-time polymerase chain reaction (qRT-PCR)

Expression of mature miRNAs was assayed using the TaqMan MicroRNA Assay in accordance with the manufacturer's instructions (Applied Biosystems). All reactions, including no-template controls and RT-minus controls, were run in an ABI Prism 7900HT Sequence detection system (Applied Biosystems). Specific RT primers and TaqMan probes were used to quantify the expression of hsa-miR-125a-3p (PN: 4395310) and hsa-miR-125a-5p (PN: 4395309). Samples were normalized to RNU6B (PN: 4373381) or U18 (PN: 4380904) as indicated. For quantification of tissue samples, RT-PCR analysis was performed in two independent experiments, each using two independent samples. For quantification of cell samples, RT-PCR analysis was performed in three independent experiments, each using three independent samples. MiRNA expression data is presented as fold difference relative to either RNU6B or U18 based on the following equation: RQ = 2^-ΔΔCt^.

### Cell migration assays

For cell migration assays, 5 × 10^4 ^cells were trypsinized, washed, resuspended in serum-free RPMI1640 or DMEM, and placed in the top portion of the chamber. The lower portion of the chamber contained 10% FBS for use as a chemoattractant. The chambers were incubated at 37°C in 5% CO_2 _for 24 h, washed with PBS, and fixed in 100% methanol. Following fixation, cells were stained with Haematoxylin and imaged, and the number of migrating cells was counted. Five random fields were analyzed for each chamber. Assays were conducted in duplicate in three independent experiments.

### Cell invasion assays

Pre-chilled serum-free RPMI1640 or DMEM was mixed with Matrigel (1:7; BD Biosciences). The upper compartments of the chambers were filled with 100 μ1 of the mixture, and the Matrigel was allowed to solidify at room temperature for 4 h. After solidification, 5 × 10^4 ^cells were trypsinized, washed, resuspended in serum-free RPMI1640 or DMEM, and placed in the top portion of the chamber. The lower portion of the chamber contained 10% FBS for use as a chemoattractant. The chambers were incubated at 37°C in 5% CO_2 _for 24 h, washed with PBS, and fixed in 100% methanol. Fixed cells were stained with Haematoxylin and imaged, and the number of invasive cells was counted. Five random fields were analyzed for each chamber. Assays were conducted in duplicate in three independent experiments.

### Statistical analysis

The SPSS 13.0 statistical software package was used for all analyses. For real-time PCR data, the statistical analysis of hsa-miR-125a-3p/5p expression levels in NSCLC tissues and corresponding LAC tissues was log2 transformed. Paired-samples T-test was used to analyze significant differences in hsa-miR-125a-3p/5p expression between NSCLC and LAC tissues. All values are expressed as mean ± SD. The Chi-square test was used to determine the relationship between hsa-miR-125a-3p/5p expression and clinicopathological variables. The two-sided Fisher's exact test to determine the relationship between hsa-miR-125a-3p/5p expression and clinicopathological variables when the number of tumors analyzed was less than 5 [21]. The Mann-Whitney test was used for pathological grade and clinical stage ranked data analysis. Spearman correlation analysis was used to determine the correlation between hsa-miR-125a-3p/5p expression and clinical stage and lymph node metastasis status. Other results were analyzed using independent samples T-test. Results were considered to be statistically significant at values of p < 0.05.

## Results

### Downregulation of hsa-miR-125a-3p/-5p expression in NSCLC tissues

**Figure 1:**
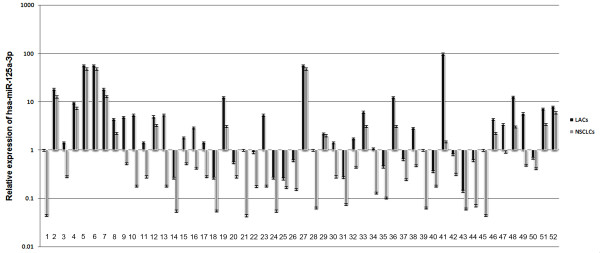
**Relative expression of hsa-miR-125a-3p in lung cancer tissues**. The relative expression of hsa-miR-125a-3p decreased in 52 NSCLCs in comparison to corresponding LACs. The data are representative of three independent experiments, and the relative expression values were calculated using the equation RQ = 2^-ΔΔCt^.

**Figure 2:**
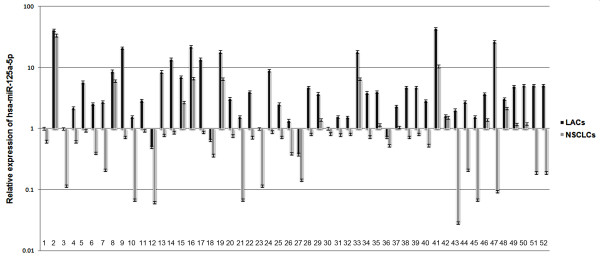
**Relative expression of hsa-miR-125a-5p in lung cancer tissues**. The relative expression of hsa-miR-125a-5p decreased in 52 NSCLCs in comparison to corresponding LACs. The results are representative of three independent experiments, and the relative expression values were calculated using the equation RQ = 2^-ΔΔCt^.

**Figure 3:**
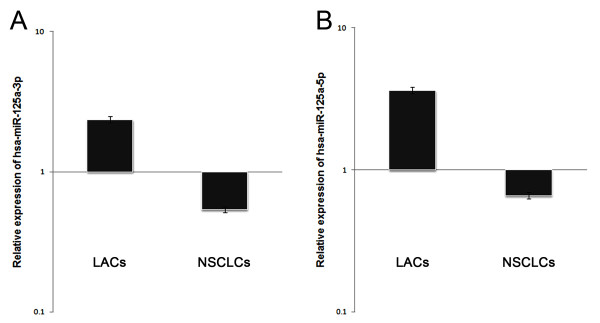
**Mean levels of hsa-miR-125a-3p and hsa-miR-125a-5p in lung cancer tissues**. (A) The mean level of hsa-miR-125a-3p relative expression in 52 NSCLCs. (B) The mean level of hsa-miR-125a-5p relative expression in 52 NSCLCs. The values represent the means of three replicates, and the relative expression values were calculated using the equation RQ = 2^-ΔΔCt^.

Although it has been observed that expression of hsa-miR-125a-5p is downregulated in NSCLC using miRNA microarray analysis, expression levels of hsa-miR-125a-3p in NSCLC remain unknown. Therefore, we examined the relative expression of hsa-miR-125a-3p and hsa-miR-125a-5p in 52 NSCLCs and paired LACs by real-time PCR. Expression of RNU6B was used as an internal standard. Expression of hsa-miR-125a-3p/5p decreased in NSCLCs in comparison to matched LACs (p < 0.05, AUC-3p = 0.735, AUC-5p = 0.845, Fig 1 and Fig 2, Additional file 1). The mean expression levels of hsa-miR-125a-3p in NSCLCs was decreased approximate 4.5 folds of that in LACs (p < 0.001, Fig 3A) and hsa-miR-125a-5p was decreased approximate 6 folds (p < 0.001, Fig 3B).

### Correlation between hsa-miR-125a-3p/5p expression and clinicopathological variables of NSCLC

To determine the effects of hsa-miR-125a-3p/5p expression on tumor initiation and progression, lung cancer patients were divided into two groups based on the mean level of hsa-miR-125a-3p and hsa-miR-125a-5p expression in 52 NSCLCs. The two groups were defined as follows: hsa-miR-125a-3p low expression and high expression (1.2352 of the log2 value) and hsa-miR-125a-5p low expression and high expression (1.8594 of the log2 value) [22]. The relationships between hsa-miR-125a-3p/5p expression and clinicopathological variables for lung cancer are shown in Table 1 and Table 2. Statistically significant associations between hsa-miR-125a-3p and hsa-miR-125a-5p expression and pathological stage were observed (p = 0.012 and p = 0.002, respectively, Mann-Whitney Test). Changes in expression of hsa-miR-125a-3p and hsa-miR-125a-5p were also statistically significantly associated with lymph node metastasis in lung cancer (p = 0.034 and p = 0.042, respectively, Two-sided Fisher's Exact Test). No correlation was observed between hsa-miR-125a-3p/5p expression and gender, histology type, or pathological grade. However, the hsa-miR-125a-3p expression level was correlated with age (p = 0.031, Chi-square Test), in contrast to that of hsa-miR-125a-5p.

**Table 1:** Relationship between hsa-miR-125a-3p expression and clinical pathological factors in lung cancinoma

		hsa-miR-125a-3p		
		
Variable	Patients	Low expression(%)	High expression(%)	p value
Gender				
Male	33	12 (36.4)	21 (63.6)	
Female	19	6 (31.6)	13 (68.4)	0.727^b^
Age				
≤ 60	28	22 (78.6)	6 (21.4)	
> 60	24	12 (50.0)	12 (50.0)	0.031^b^
Histology type				
Squamous cancer	22	13 (59.1)	9 (40.9)	
Adenocarcinoma	30	21 (70.0)	9 (30.0)	0.414^b^
Pathological grade				
I	8	7 (87.5)	1 (12.5)	
II	28	17 (60.7)	11 (39.3)	
III	16	10 (62.5)	6 (37.5)	0.370^c^
Pathological stage				
I	18	8 (44.4)	10 (55.6)	
II	15	10 (66.7)	5 (33.3)	
III	19	16 (80.0)	3 (20.0)	0.012^c^
Lymph node status				
No metastasis	32	17 (53.1)	15 (46.9)	
Metastasis	20	17 (85.0)	3 (15.0)	0.034^a^

^a ^Two-sided Fisher's Exact Test.

^b ^Chi-square Test

^c ^Mann-whitney Test

**Table 2:** Relationship between hsa-miR-125a-5p expression and clinical pathological factors in lung cancinoma

		hsa-miR-125a-5p		
		
Variable	Patients	Low expression(%)	High expression(%)	p value
Gender				
Male	33	14 (42.4)	19 (57.6)	
Female	19	6 (31.6)	13 (68.4)	0.439^b^
Age				
≤ 60	28	11 (39.3)	17 (60.7)	
> 60	24	9 (37.5)	15 (62.5)	1.000^b^
Histology type				
Squamous cancer	22	9 (40.9)	13 (59.1)	
Adenocarcinoma	30	11 (36.7)	19 (63.3)	0.780^b^
Pathological grade				
I	8	4 (50.0)	4 (50.0)	
II	28	11 (39.3)	17 (60.7)	
III	16	5 (31.3)	11 (68.7)	0.380^c^
Pathological stage				
I	18	12 (66.7)	6 (33.3)	
II	15	5 (33.3)	10 (66.7)	
III	19	3 (15.8)	16 (84.2)	0.002^c^
Lymph node status				
No metastasis	32	16 (50.0)	16 (50.0)	
Metastasis	20	4 (20.0)	16 (80.0)	0.042^a^

^a ^Two-sided Fisher's Exact Test.

^b ^Chi-square Test

^c ^Mann-whitney Test

In order to better characterize the relationship between hsa-miR-125a-3p/5p expression and pathological stage and lymph node metastasis, we further analyzed the pathological stage and lymph node metastasis data using the Spearman correlation test. The results showed a negative correlation between hsa-miR-125a-3p expression and pathological stage (r = -0.352, p = 0.011), as well as lymph node metastasis (r = -0.326, p = 0.018). However, the correlations between hsa-miR-125a-5p expression and pathological stage (r = 0.439, p = 0.001) and lymph node metastasis (r = 0.300, p = 0.031) were positive.

### Predicted and confirmed mRNA targets

**Figure 4:**
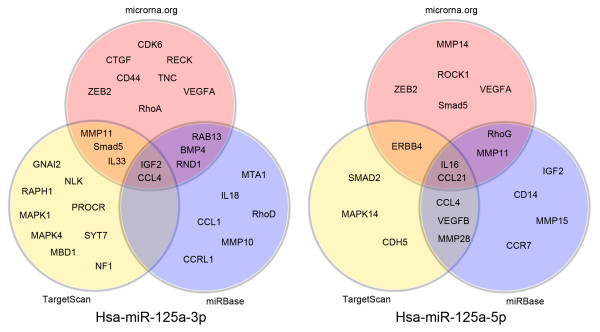
**Prediction of hsa-miR-125a-3p and hsa-miR-125a-5p target genes**. Predictions were conducted using microrna.Org, TargetScanHuman 5.1, and MiRBase webservers. Microrna.org and MiRBase predictions are based on the miRanda algorithm, while TargetScanHuman5.1 predictions are based on the TargetScan algorithm.

Results from tissue expression analysis indicated that hsa-miR-125a-3p and hsa-miR-125a-5p are associated with metastasis. Thus, we used computational analysis to predict metastasis-related target mRNAs of hsa-miR-125a-3p and hsa-miR-125a-5p. The analysis was conducted using microrna.org and the TargetScanHuman 5.1 and MiRBase webservers. Target predictions from Microrna.org and MiRBase are based on the miRanda algorithm. TargetScanHuman5.1 utilized the TargetScan algorithm. The relationships between hsa-miR-125a-3p/5p and their potential target genes identified from these three databases are shown in Fig.4, Additional file 2, and Additional file 3. The details of these target genes are shown in Additional file 4 and Additional file 5.

### Downregulation of hsa-miR-125a-3p/5p expression in NSCLC cell lines

**Figure 5:**
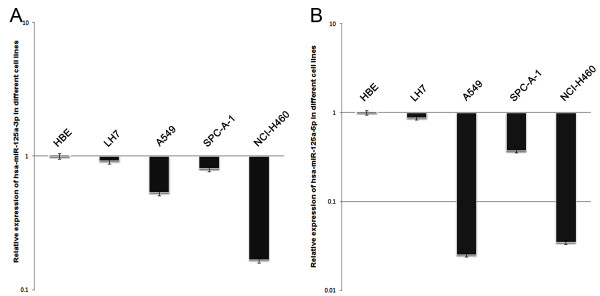
**Relative expression of hsa-miR-125a-3p and hsa-miR-125a-5p in different lung cancer cell lines**. (A) The relative expression levels of hsa-miR-125a-3p in four lung cancer cell lines, particularly the NCI-H460 cell line, were all lower than that of the HBE cell line. (B) The relative expression levels of hsa-miR-125a-5p in four lung cancer cell lines, particularly the A459 cell line, were all lower than that of the HBE cell line. The results are representative of three independent experiments, and the relative expression values were calculated using the equation RQ = 2^-ΔΔCt^.

In order to identify suitable cell lines for further studies, we examined the relative expression levels of hsa-miR-125a-3p/5p in four lung cancer cell lines (LH7, A549, SPC-A-1, and NCI-H460). Expression of hsa-miR-125a-3p/5p in the lung cancer cell lines was normalized to that of a control human bronchiolar epithelium (HBE) cell line. U18 was used as an internal standard for real-time PCR. We found that the expression levels of hsa-miR-125a-3p and hsa-miR-125a-5p were lower in each of the four lung cancer cell lines than in the HBE cell line. The mean level of hsa-miR-125a-3p expression was moderately decreased in A549 cells (p < 0.001, Fig 5A), and the mean level of hsa-miR-125a-5p expression was moderately decreased in SPC-A-1 cells (p < 0.001, Fig 5B). Therefore, we chose the A549 cell line for further studies regarding hsa-miR-125a-3p and the SPC-A-1 cell line for further studies regarding hsa-miR-125a-5p.

### Effects of gain-of-function of hsa-miR-125a-3p/5p on migratory and invasive capabilities of A549 and SPC-A-1 cells

**Figure 6:**
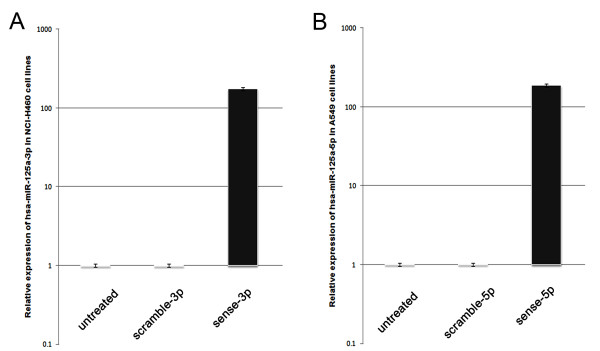
**Relative levels of hsa-miR-125a-3p and hsa-miR-125a-5p were increased by transfection with sense 2'-O-methyl oligonucleotides**. (A) The relative levels of hsa-miR-125a-3p were significantly increased in the sense-3p-transfected group. (B) The relative levels of hsa-miR-125a-5p were significantly increased in the sense-5p-transfected group. The results are representative of three independent experiments, and the relative expression values were calculated using the equation RQ = 2^-ΔΔCt^.

To determine whether hsa-miR-125a-3p and hsa-miR-125a-5p are associated with migration and invasion in lung cancer cells, we adopted a gain-of-function approach. The quantities of hsa-miR-125a-3p/5p were exogenously increased by transfection of sense 2'-O-methyl oligonucleotides into A549/SPC-A-1 cells. We first utilized real-time PCR to analyze the expression of hsa-miR-125a-3p/5p to ensure that transfection with sense 2'-O-methyl oligonucleotides increased its expression after 24 hours. Expression in transfected cells was normalized to that of untreated cells, and U18 expression was used as an internal standard. The results showed that, in comparison to the control untreated groups, the mean levels of expression of both hsa-miR-125a-3p and hsa-miR-125a-5p in the sense groups were significantly increased (p < 0.001 for both, Fig 6A and 6B).

**Figure 7:**
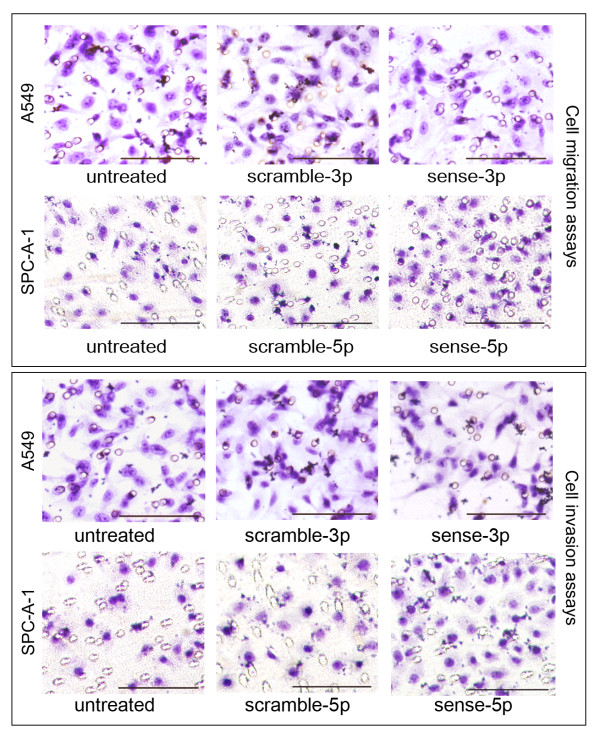
**Effects of gain-of-function of hsa-miR-125a-3p/5p on migration and invasion of lung cancer cells**. Migration assay results showed that the number of A549 cells that migrated through the microporous membrane was significantly decreased in the sense-3p group (p < 0.001). In contrast, the number of migratory SPC-A-1 cells was significantly increased in the sense-5p group (p < 0.001, bar = 20 μm). Invasion assay results showed that the number of A549 cells that invaded through the Matrigel was significantly decreased in the sense-3p group (p < 0.001). However, the number of invasive SPC-A-1 cells significantly increased in the sense-5p group (p < 0.001, bar = 20 μm). The results are representative of three independent experiments.

We next analyzed the migratory capabilities of cells overexpressing hsa-miR-125a-3p/5p using transwell chambers. For analysis of hsa-miR-125a-3p, the number of A549 cells in the untreated group that migrated through a microporous membrane was 31.20 ± 1.64. There was no difference between untreated cells and cells transfected with the scramble-3p oligonucleotide (31.40 ± 0.55, p = 0.807). However, the number of migrating cells was significantly decreased when cells were transfected with the sense-3p oligonucleotide (16.40 ± 2.07, p < 0.001). For hsa-miR-125a-5p, the number of SPC-A-1 cells in the untreated group that migrated through the microporous membrane was 26.60 ± 2.07. Again no difference was observed between untreated cells and cells transfected with the scramble-5p oligonucleotide (25.60 ± 1.67, p = 0.426). However, the number of migrating cells increased significantly when cells were transfected with the sense-5p oligonucleotide (38.80 ± 1.92, p < 0.001, Fig 7).

In the hsa-miR-125a-3p invasion analysis, the number of A549 cells in the untreated group that invaded through the Matrigel was 24.40 ±2.41. There was no difference between untreated cells and cells transfected with the scramble-3p oligonucleotide (24.80 ± 3.03, p = 0.823). The number of invading cells decreased significantly when cells were transfected with the sense-3p oligonucleotide (12.20 ± 1.58, p < 0.001). In the invasion assay for hsa-miR-125a-5p, the number of SPC-A-1 cells in the untreated group that invaded through the Matrigel was 22.20 ± 1.72. Again no significant difference was observed between untreated cells and cells transfected with the scramble-5p oligonucleotide (23.40 ± 2.34, p = 1.000). However, the number of invading cells increased significantly when cells were transfected with the sense-5p oligonucleotide (30.80 ± 1.92, p < 0.001, Fig 7).

### Effects loss-of-function of hsa-miR-125a-3p/5p on the migratory and invasive capabilities of A549 and SPC-A-1 cells

**Figure 8:**
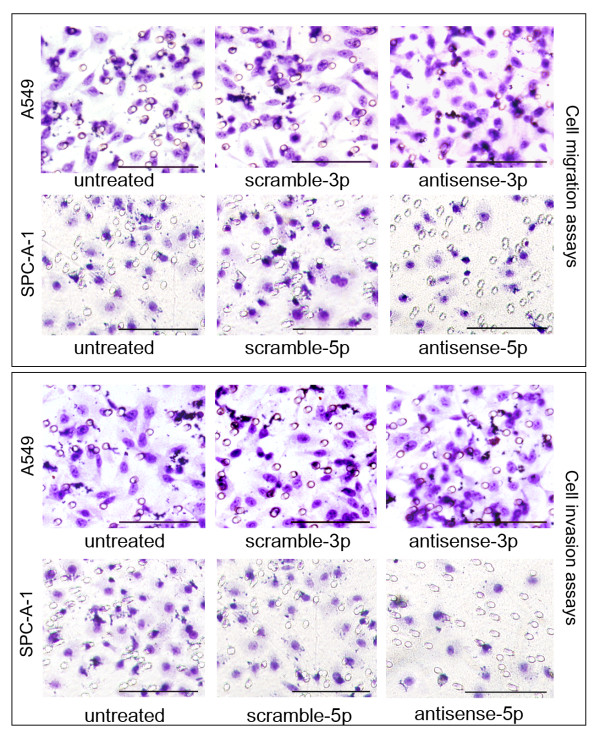
**Effects of loss-of-function of hsa-miR-125a-3p/5p on migration and invasion of lung cancer cells**. Migration assay results showed that the number of A549 cells that migrated through the microporous membrane was significantly increased in the antisense-3p group (p < 0.001). However, the number of migratory SPC-A-1 cells was significantly decreased in the antisense-5p group (p < 0.001, bar = 20 μm). Invasion assay results showed that the number of A549 cells that invaded through the Matrigel was significantly increased in the antisense-3p group (p < 0.001). In contrast, the number of invasive SPC-A-1 cells was significantly decreased in the antisense-5p group (p < 0.001, bar = 20 μm). The results are representative of three independent experiments.

To further examine whether endogenous hsa-miR-125a-3p and hsa-miR-125a-5p regulate migration and invasion, we adopted a loss-of-function approach that blocked the function of endogenous hsa-miR-125a-3p and hsa-miR-125a-5p using antisense 2'-O-methyl oligonucleotides [23-26]. We then analyzed the migratory and invasive capabilities of these cells using the methods described above. In the hsa-miR-125a-3p migration assay, the number of A549 cells in the untreated group that migrated through the microporous membrane was 31.00 ± 1.54. There was no difference between the untreated and scramble-3p-transfected cells (32.00 ± 0.58, p = 0.347). The number of migrating cells was significantly increased when A549 cells were transfected with the antisense-3p oligonucleotide (46.00 ± 3.16, p < 0.001). For the hsa-miR-125a-5p migration assay, the number of SPC-A-1 cells in the untreated group that migrated through the microporous membrane was 22.60 ± 2.07. No difference was observed between untreated and scramble-5p-transfected cells (22.40 ± 1.14, p = 0.855). The number of migrating cells was significantly decreased when SPC-A-1 cells were transfected with the antisense-5p oligonucleotide (15.00 ± 1.58, p < 0.001, Fig 8).

In the hsa-miR-125a-3p invasion assay, the number of A549 cells in the untreated group that invaded through the Matrigel was 25.00 ± 1.51. There was no difference between untreated and scramble-3p-transfected cells (24.60 ± 3.04, p = 0.801). The number of invading cells was significantly increased when A549 cells were transfected with the antisense-3p oligonucleotide (37.00 ± 2.73, p < 0.001). In the hsa-miR-125a-5p invasion assay, the number of SPC-A-1 cells in the untreated group that invaded through the Matrigel was 21.00 ± 1.59. No significant difference was observed between untreated and scramble-5p-transfected cells (20.80 ± 0.84, p = 0.809). The number of invading SPC-A-1 cells decreased significantly when cells were transfected with the antisense-5p oligonucleotide (11.00 ± 1.07, p < 0.001, Fig 8).

## Discussion

MiRNAs have emerged as important regulators of tumorigenesis [27,28]. Mounting evidence has shown that miRNAs are frequently mutated or differentially expressed in human cancers, suggesting that miRNAs may act as tumor suppressors or oncogenes depending on whether their specific targets act as oncogenes or tumor suppressors [29]. Therefore, tumor suppressor miRNAs are generally expressed at low levels [30,31] and oncogenic miRNAs are expressed at high levels in tumors or tumor cell lines [32,33]. These observations suggest that specific characterization of miRNA expression and function could lead to the identification of novel biomarkers to predict clinical outcomes in the future [34-37].

In the present study, we report that expression of hsa-miR-125a-3p and hsa-miR-125a-5p is dysregulated in lung cancer. Consistent with the results of Yanainhara [20], real-time PCR results showed that expression of hsa-miR-125a-5p was decreased significantly in the majority of NSCLCs in comparison to matched LACs. In addition, we also found that expression of hsa-miR-125a-3p was significantly reduced. This expression pattern suggests that hsa-miR-125a-3p and hsa-miR-125a-5p may act as tumor suppressors by regulating expression of a subset of oncogenes. These specific alterations in expression may be characteristic of human lung cancer and may contribute to lung tumorigenesis.

Tumor invasion and metastasis are the most common cause of cancer deaths worldwide [38,39]. Studies regarding the functions of miRNAs in tumor invasion and metastasis currently represent a major focus in cancer biology [14,40]. In this study, we evaluated the correlation between hsa-miR-125a-3p/5p expression levels and clinicopathological variables in 52 NSCLC tissues. Interestingly, we found that hsa-miR-125a-3p/5p expression levels were correlated with pathological stage and lymph node metastasis. Together with our functional studies in lung cancer cells, these results suggest that hsa-miR-125a-3p and hsa-miR-125a-5p function in opposite manners to suppress and enhance cell migration and invasion of lung cancer cells, respectively, which may in turn affect lymph node metastasis in NSCLC.

The specific mechanisms that lead to changes in cell migration and invasion regulated by hsa-miR-125a-3p/5p in lung cancer cells remain unknown. MiRNAs are commonly thought to posttranscriptionally regulate mRNA degradation and inhibition of mRNA translation via interactions with the 3'-untranslated regions (UTRs) of target mRNAs in animals [16-18]. However, there are several remaining gaps in our knowledge of miRNA function that have been revealed by recent studies. Recently, a few experiments have indicated possible target sites in the 5'-UTR of mRNAs [41]. Furthermore, 0rom and colleagues [7] found that miRNA-10a binds the 5'UTR of ribosomal protein mRNAs and enhances their translation. Therefore, the regulatory mechanisms controlled by miRNAs are may be more varied and extensive than previously thought.

According to predicted results from three webserver databases, CCL4 and IGF-2 were the most likely target genes of hsa-miR-125a-3p, while IL16 and CCL21 were the most likely target genes of hsa-miR-125a-5p. CCL4 (MIP-1beta), a member of the CC chemokine family, induces cellular migration and invasion through interactions with its receptor CCR5 [42,43]. Nussbaum and colleagues [44] have reported that IGF-2 and its receptor IGF-2R stimulate tumor cell migration in human hepatocarcinogenesis. IL-16, an activator of the plasminogen-plasmin system, promotes human eosinophil migration into the extracellular matrix via a CCR3-chemokine-mediated signaling pathway [45]. Previous results from our laboratory indicated that CCR7 and its ligand CCL21 regulate migration and invasion of lung cancer cells through the ERK1/2 pathway under hypoxic conditions and promote metastasis of lung cancer [46]. Therefore, the complicated regulatory mechanisms controlled by hsa-miR-125a-3p and hsa-miR-125a-5p may play complex roles in migration and invasion. Despite the necessity for further studies to understand these mechanisms, our findings suggest a crucial role for miR-125a in lung cancer.

## Conclusion

The results from this study demonstrate that hsa-miR-125a-3p and hsa-miR-125a-5p, two miRNAs that are downregulated in NSCLC, are associated with lymph node metastasis. *In vitro *functional studies in lung cancer cells revealed that hsa-miR-125a-3p and hsa-miR-125a-5p appear to play opposite cellular roles, suppressing and enhancing cell migration and invasion, respectively. Thus, these results may lead to the identification of novel biomarkers and therapeutic strategies to combat lymph node metastasis.

## Competing interests

The authors declare that they have no competing interests.

## Authors' contributions

LJ initiated the research, carried out the experiments, and wrote the manuscript. QH and SZ contributed to translation of the paper. QZ and JC provided experimental instruction. XQ helped with the experimental design and provided funding support. EW provided critical review of the manuscript. All authors read and approved the final manuscript.

## Pre-publication history

The pre-publication history for this paper can be accessed here:

http://www.biomedcentral.com/1471-2407/10/318/prepub

## Supplementary Material

Additional file 1 **The specificity and sensitivity of hsa-miR-125a-3p and hsa-miR-125a-5p**. (A) The specificity and sensitivity of hsa-miR-125a-3p was analyzed by DOC Curve. (B) The specificity and sensitivity of hsa-miR-125a-5p was analyzed by DOC Curve. Click here for file

Additional file 2 **Prediction of target sites for hsa-miR-125a-3p and target mRNAs in three databases**. The target sites for hsa-miR-125a-3p and the target mRNAs were predicted using microrna.org, TargetScanHuman 5.1 and MiRBase webservers to further illustrate results shown in Fig. 4. Click here for file

Additional file 3 **Prediction of target sites for hsa-miR-125a-5p and target mRNAs in three databases**. The target sites of hsa-miR-125a-5p and the target mRNAs were predicted using microrna.org, TargetScanHuman 5.1 and MiRBase webservers to further illustrate results shown in Fig. 4. Click here for file

Additional file 4 **Details of the target genes of hsa-miR-125a-3p**. Details of the target genes of hsa-miR-125a-3p. Click here for file

Additional file 5 **Details of the target genes of hsa-miR-125a-5p**. Details of the target genes of hsa-miR-125a-5p. Click here for file
