# Supplementary figures and images for: Hsa-miR-125a-3p and hsa-miR-125a-5p are downregulated in non-small cell lung cancer and have inverse effects on invasion and migration of lung cancer cells
Source: BMC Cancer. 2010 Jun 22;10:318. doi: 10.1186/1471-2407-10-318 (PMC2903529; doi:10.1186/1471-2407-10-318)

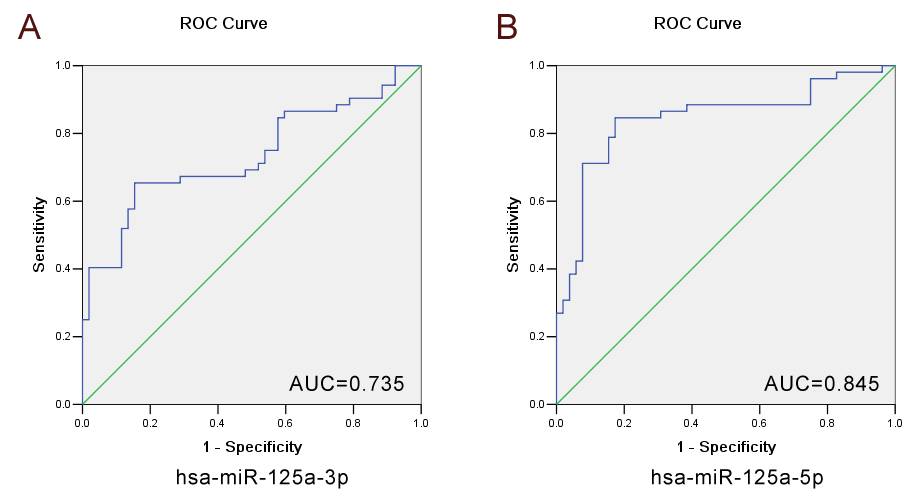

Supplement: Additional file 1 — The specificity and sensitivity of hsa-miR-125a-3p and hsa-miR-125a-5p. (A) The specificity and sensitivity of hsa-miR-125a-3p was analyzed by DOC Curve. (B) The specificity and sensitivity of hsa-miR-125a-5p was analyzed by DOC Curve. [file 1471-2407-10-318-S1.TIFF]

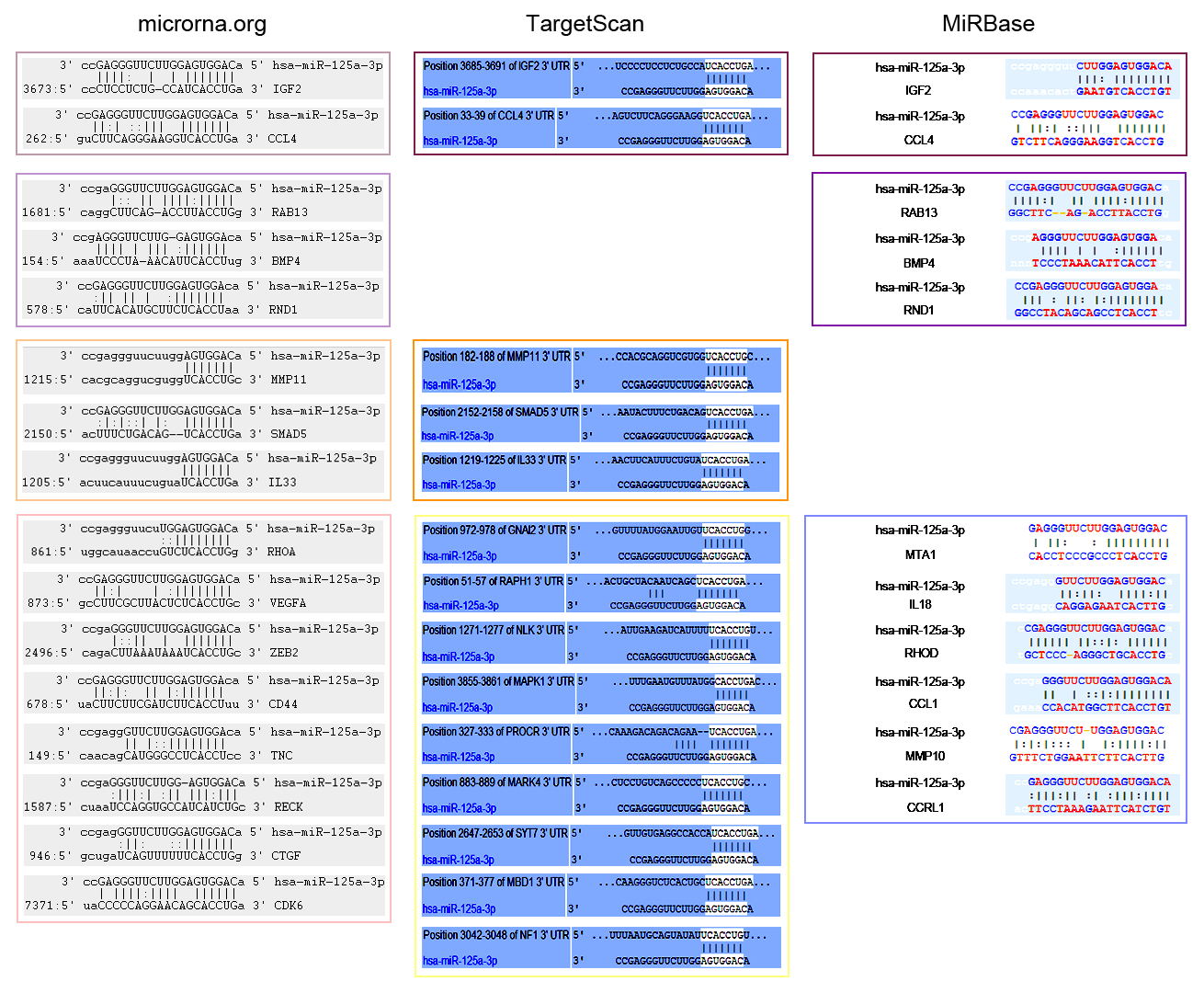

Supplement: Additional file 2 — Prediction of target sites for hsa-miR-125a-3p and target mRNAs in three databases. The target sites for hsa-miR-125a-3p and the target mRNAs were predicted using microrna.org, TargetScanHuman 5.1 and MiRBase webservers to further illustrate results shown in Fig. 4. [file 1471-2407-10-318-S2.TIFF]

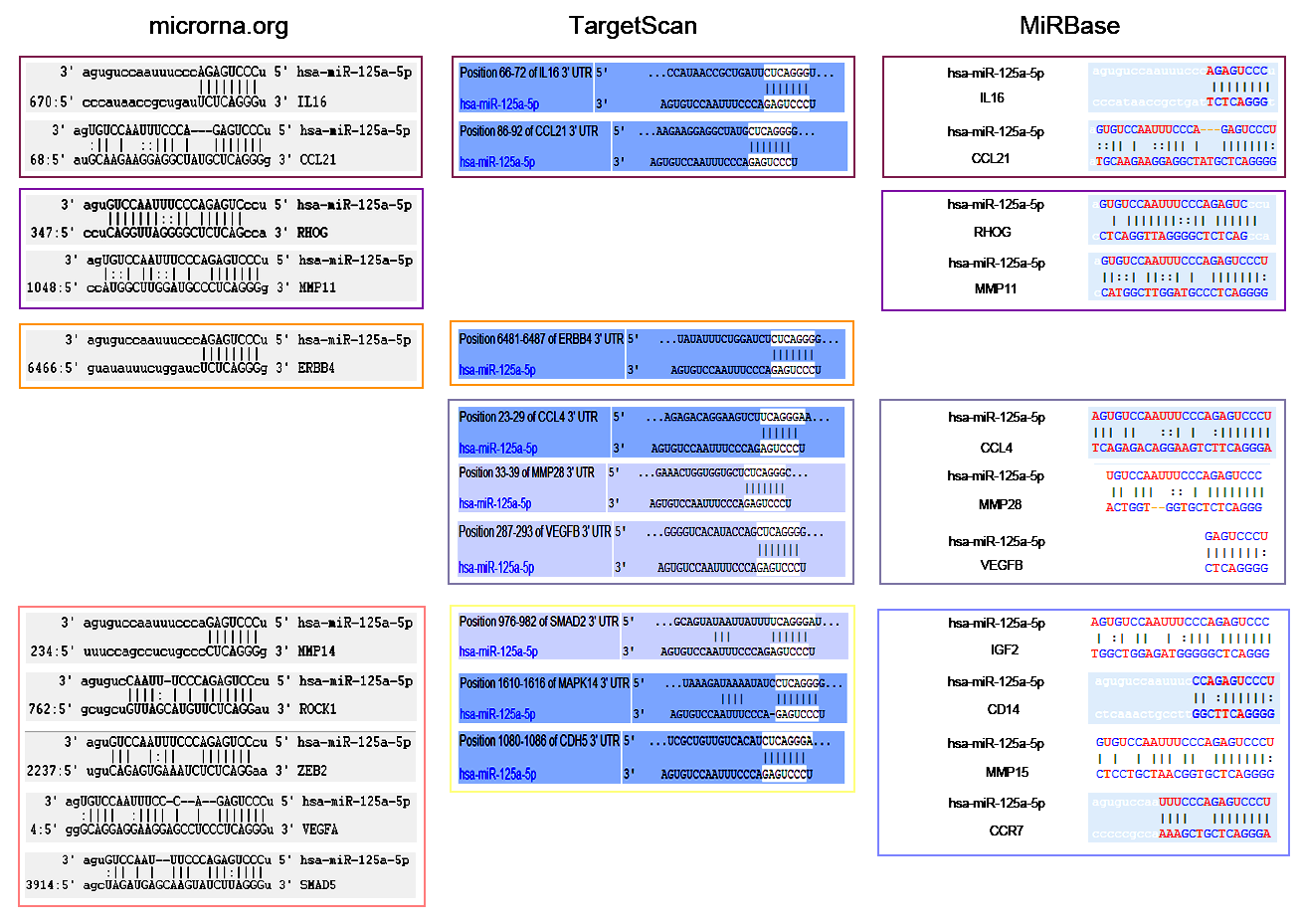

Supplement: Additional file 3 — Prediction of target sites for hsa-miR-125a-5p and target mRNAs in three databases. The target sites of hsa-miR-125a-5p and the target mRNAs were predicted using microrna.org, TargetScanHuman 5.1 and MiRBase webservers to further illustrate results shown in Fig. 4. [file 1471-2407-10-318-S3.TIFF]
