# Supplementary material for: Hsa-miR-125a-3p and hsa-miR-125a-5p are downregulated in non-small cell lung cancer and have inverse effects on invasion and migration of lung cancer cells
Source: BMC Cancer. 2010 Jun 22;10:318. doi: 10.1186/1471-2407-10-318 (PMC2903529; doi:10.1186/1471-2407-10-318)
Supplement: Additional file 4 — Details of the target genes of hsa-miR-125a-3p. Details of the target genes of hsa-miR-125a-3p. [file 1471-2407-10-318-S4.PDF]

Additional file 4: Details of the target genes of hsa-miR-125a-3p

Gene Name		Function
IGF2	insulin-like growth factor 2	proliferation, differentiation, invasion
CCL4	chemokine (C-C motif) ligand 4	migration, invasion
MMP11	matrix metallopeptidase 11	chemotactic influence on macrophages, invasion
Smad5	SMAD family member 5	proliferation, differentiation, migration, apoptosis
IL33	interleukin 33	inflammation, migration, adhesion
RAB13	member RAS oncogene family	membrane trafficking, cell scattering
BMP4	bone morphogenetic protein 4	proliferation, differentiation, invasion, apoptosis, epithelial-mesenchymal transition(EMT)
RND1	Rho family GTPase 1	organization of the actin cytoskeleton
GNAI2	guanine nucleotide binding protein (G protein), alpha inhibiting activity polypeptide 2	hormonal regulation of adenylate cyclase, invasion
NLK	nemo-like kinase	proliferation, migration, invasion
RAPH1	Ras association (RalGDS/AF-6) and pleckstrin homology domains 1	cytoskeletal rearrangement, invasion
MAPK1	mitogen-activated protein kinase 1	proliferation, differentiation, apoptosis, migration
MAPK4	mitogen-activated protein kinase 4	proliferation, differentiation, apoptosis, migration
MBD1	methyl-CpG binding domain protein 1	proliferation, apoptosis, invasion and migration
PROCR	protein C receptor, endothelial (EPCR)	cytoskeletal rearrangement, migration
SYT7	synaptotagmin VII	invasion
NF1	neurofibromin 1	differentiation, proliferation
MTA1	metastasis associated 1	migration, invasion
IL18	interleukin 18	inflammation, apoptosis, migration
RhoD	ras homolog gene family, member D	cytoskeletal reorganization, migration
CCL1	chemokine (C-C motif) ligand 1	inflammation, migration

MMP10	matrix metallopeptidase 10	proliferation, invasion
CCRL1	chemokine (C-C motif) receptor-like 1	invasion
CDK6	cyclin-dependent kinase 6	proliferation, migration, invasion
CTGF	connective tissue growth factor	differentiation, proliferation, migration, invasion, EMT
ZEB2	zinc finger E-box binding homeobox 2	EMT, migration, invasion
RhoA	ras homolog gene family, member A	adhesion, chemotaxis, EMT, migration, invasion
RECK	reversion-inducing-cysteine-rich protein with kazal motifs	adhesion, migration, invasion
TNC	tenascin C	proliferation, migration, invasion, inflammation
VEGFA	vascular endothelial growth factor A	migration, differentiation, synaptogenesis, myelination
