# Supplementary material for: Hsa-miR-125a-3p and hsa-miR-125a-5p are downregulated in non-small cell lung cancer and have inverse effects on invasion and migration of lung cancer cells
Source: BMC Cancer. 2010 Jun 22;10:318. doi: 10.1186/1471-2407-10-318 (PMC2903529; doi:10.1186/1471-2407-10-318)
Supplement: Additional file 5 — Details of the target genes of hsa-miR-125a-5p. Details of the target genes of hsa-miR-125a-5p. [file 1471-2407-10-318-S5.PDF]

Additional file 5: Details of the target genes of hsa-miR-125a-5p

Gene Name		Function
IL16	interleukin 16	migration, inflammation
CCL21	chemokine (C-C motif) ligand 21	migration, inflammation
ERBB4	v-erb-a erythroblastic leukemia viral oncogene homolog 4	proliferation, differentiation, apoptosis, migration, invasion
RhoG	ras homolog gene family, member G	migration
MMP11	matrix metallopeptidase 11	chemotactic influence on macrophages, invasion
CCL4	chemokine (C-C motif) ligand 4	migration, invasion
VEGFB	vascular endothelial growth factor B	differentiation, migration, invasion
MMP28	matrix metallopeptidase 28	adhesion, proliferation, migration
MMP14	matrix metallopeptidase 14	proliferation, invasion
ROCK1	Rho-associated, coiled-coil containing protein kinase 1	apoptosis, cytoskeletal reorganization, EMT, migration, invasion
ZEB2	zinc finger E-box binding homeobox 2	EMT, migration, invasion
Smad5	SMAD family member 5	proliferation, differentiation, migration, apoptosis
VEGFA	vascular endothelial growth factor A	migration, differentiation, synaptogenesis, myelination
Smad2	SMAD family member 2	proliferation, apoptosis, cytoskeletal reorganization, cell motility, EMT
MAPK14	mitogen-activated protein kinase 14	proliferation, differentiation, apoptosis, migration
CDH5	cadherin 5	adhesion, proliferation, migration
IGF2	insulin-like growth factor 2	proliferation, differentiation, invasion
CD14	CD14 molecule	inflammation
MMP15	matrix metallopeptidase 15	migration, invasion
CCR7	chemokine (C-C motif) receptor 7	chemotaxis, proliferation, apoptosis, migration, invasion
